# Non-Skeletal Activities of Vitamin D: From Physiology to Brain Pathology

**DOI:** 10.3390/medicina55070341

**Published:** 2019-07-05

**Authors:** Giulia Bivona, Luisa Agnello, Chiara Bellia, Giorgia Iacolino, Concetta Scazzone, Bruna Lo Sasso, Marcello Ciaccio

**Affiliations:** 1 Institute of Clinical Biochemistry, Clinical Molecular Medicine and Laboratory Medicine, Department of Biomedicine, Neuroscience and Advanced Diagnostics, University of Palermo, 90127 Palermo, Italy; 2 Department and U.O.C. Laboratory Medicine, University Hospital “Paolo Giaccone” of Palermo, 90127 Palermo, Italy

**Keywords:** vitamin D, immune system, brain function, 25(OH)D_3_, Alzheimer’s disease, Parkinson’s disease

## Abstract

Vitamin D is a secosteroid hormone regulating the expression of almost 900 genes, and it is involved in the regulation of calcium and phosphate metabolism, immune response, and brain development. Low blood vitamin D levels have been reported in patients affected by various diseases. Despite a large amount of literature data, there is uncertainty surrounding the role of vitamin D as a serum biomarker in Alzheimer’s disease (AD) and Parkinson’s disease (PD). Indeed, the lack of internationally recognized 25(OH)D_3_ reference measurement procedures and standard materials in the past led to unstandardized serum total 25(OH)D_3_ results among research and clinical care laboratories. Thus, most of the literature studies reported unstandardized data, which are of little use and make it difficult to draw conclusions of the role of vitamin D in AD and PD. This review summarizes the extra-skeletal actions of vitamin D, focusing its role in immunomodulation and brain function, and reports the issue of lacking standardized literature data concerning the usefulness of vitamin D as a biomarker in AD and PD.

## 1. Introduction

In the early 1920s, amid the industrial revolution, McCollum discovered that fish oil containing a high amount of vitamin D could treat rickets [1]. Since then, skeletal health and bone metabolism have been invariably associated with vitamin D status. Nonetheless, over the past two decades, the literature in the field of vitamin D grew fast, showing a multifaceted role of the nutrient in modulating many processes of body homeostasis. Vitamin D is regarded as a neurosteroid regulating immunomodulation and brain development and function in adulthood [2,3]. Vitamin D has been long studied in several pathologies either as a risk factor for disease development or a disease severity biomarker. However, there is no consensus on the optimal vitamin D status due to the lack of standardized measurement procedures and materials, as shown for other analytes [4,5,6]. The current paper summarizes the extra-skeletal actions of vitamin D, focusing on the modulation of immune response and brain activities. Further, it mentions the issue of the lack of vitamin D standardized data in the literature, especially concerning neurodegenerative diseases.

## 2. Vitamin D Synthesis and Metabolism

Vitamin D includes two natural compounds: Vitamin D2, which is obtained by the diet, and vitamin D3, which derives both from the diet and endogenous synthesis. Ultraviolet B rays (295–310 nm) transform the cutaneous precursor 7-dehydrocholesterol into vitamin D3 (cholecalciferol), which requires two sequential hydroxylations to form the active vitamin D3. The first hydroxylation produces 25(OH)D_3_ in the liver through the action of 25-hydroxylase. The second hydroxylation is carried out by 1-α-hydroxylase forming 1,25(OH)_2_D in the kidney, prostate, placenta, lung, brain, and immune cells, all of which express 1-α-hydroxylase [7]. It is worth mentioning that kidney 1-α-hydroxylase is regulated by parathyroid hormone (PTH) [8], and active vitamin D produced plays a crucial role in calcium/phosphorus homeostasis, while the extra-renal enzyme is tightly regulated by pro-inflammatory cytokines [9], and active vitamin D produced regulates cell proliferation and differentiation in many tissues and organs as well as immune cells [10]. All enzymes participating in vitamin D metabolism belong to the cytochrome P450 (CYP450) family, which is also involved in liver drug catabolism and oxidation/reduction reactions [11]. Other pathways of vitamin D activation are initiated by CYP11A1, leading to the production of non- or low-calcemic actions and lumisterol activation [12,13,14,15]. CYP11A1 is known to catalyze the first step of steroidogenesis, converting cholesterol to pregnenolone, but 7-dehydrocholesterol, ergosterol, lumisterol 3, and vitamins D3 and D2 are substrates for this enzyme as well.

Vitamin D receptors encompass a nuclear receptor, vitamin D Receptor (VDR), and a surface receptor, membrane-associated rapid response steroid binding (MARRS), also known as protein disulfide isomers family A members 3 (PDIA3). Other vitamin D receptors include RORα and RORγ, which are expressed in the skin, and aryl hydrocarbon receptor (AhR) [16,17,18].

Upon binding both VDR and MARRS, genomic and non-genomic actions are carried out by 1,25(OH)_2_D. The former include genes transcription activation (cathelicidin, nerve growth factor—NGF) and suppression (1-α- hydroxylase, PTH), the latter comprise the regulation of the activity of some proteins (p38 MAP kinase, protein kinase C, adenylyl cyclase, phospholipase C) and the intracellular Ca^2+^ influx [19,20]. The interaction between 1,25(OH)_2_D and PDIA3 receptor is deemed to mediate vitamin D brain action, while the modulation of the immune response depends on the interaction with VDR [21].

## 3. Vitamin D Immunomodulatory Activities

Active vitamin D modulates the immune response by interacting with innate and adaptive immune system cells, regulating the expression of cytokines, since macrophages, dendritic cells, and activated B and T lymphocytes express 1α-hydroxylase and VDR [22]. NF-kB activity is inhibited by 1,25(OH)_2_D in lymphocytes, NF-kB being a transcription factor involved in pro-inflammatory cytokines synthesis [23,24]. The antigen presenting ability of dendritic cells, as well as their survival, is diminished by vitamin D [25,26]. Importantly, T-helper (Th) cells’ balance is influenced by vitamin D. Th cells include Th1, Th2, and Th17. Vitamin D inhibits the production of Th1 and Th17 cytokines (IFN-γ, TNF-α, IL-17, IL-21, IL-12, IL-1, IL-2, IL-23, and IL-17) and increases Th2 cytokines synthesis (IL-10, IL-4), thus enhancing Th2 cells differentiation [27]. Further, active vitamin D fosters the differentiation of T-regulatory (Treg) cells, increasing the production of Treg cytokines (including FoxP3) [10]. By modulating Th cells balance and enhancing the development of Treg, active vitamin D contributes to protection against pathogens [28].

Finally, active vitamin D induces the production of antimicrobial peptides, including cathelicidin and defensin [29]. Overall, the immunomodulatory action of vitamin D ends in an increase of the innate immune response antimicrobial activity and the decrease of the adaptive immune response proinflammatory action.

## 4. Vitamin D as a Light-Dependent Control System of Immunity

The action of vitamin D on immune response should be interpreted within the context of body’s homeostatic regulation, taking into account the role of the interactions between neuroendocrine and immune systems in regulating such homeostatic balance [30,31]. In this scenario, the operation of the three main mechanisms controlling the immune response, including vitamin D, vagus nerve activity, and melatonin, has emerged [32,33]. Within the context of homeostatic regulation, a significant role is played by circadian rhythms, which depend on many tissue-specific, cellular clocks, and is strictly influenced by exogenous rhythms, like light-dark rhythm, due to a synchronization operated by the suprachiasmatic nucleus of the hypothalamus [34]. Research in the field of circadian rhythm has shown that this is related to the immune response [34]. It has been suggested that each of these immune response control systems could be a part of a light-dependent immunity regulation system [35]. Indeed, light inhibits both melatonin production and the activity of vagus nerve via suprachiasmatic nucleus, while inducing the production of vitamin D. While both melatonin and vagus nerve reach the peak of expression and activity in the night, the maximum vitamin D synthesis is obtained during the day. This might suggest that the immune response control systems work alternatively in a light-dependent manner, assuring a controlled immune system activity cyclically over 24 h.

## 5. Vitamin D and Cerebral Activity

Many areas of the brain, including amygdala, hippocampus, thalamus, cortex, and substantia nigra, express both VDR and 1α-hydroxylase [36]. Multiple lines of evidence show that vitamin D can be actively synthesized by neurons and microglia, which use the active hormone to regulate cell proliferation, differentiation, and survival [37]. It has been documented that vitamin D can influence fundamental processes for brain development in the embryonic brain, including synaptic plasticity and cytoskeleton maintenance [38,39]. Almeras et al. demonstrated that prenatal vitamin D deficiency alters the expression of drebrin and growth-associated protein-43 (GAP-43), two synaptic plasticity-related proteins whose alterations have been reported in schizophrenia [40]. Drebrin is an actin-binding protein being present in both the developmental and adult brain in two isoforms, drebrin E and drebrin A, respectively. Drebrin A expression correlates to synapse formation, and its dysregulation could give a reason for dendritic spine alteration observed in schizophrenia patients [41]. GAP-43 controls axonal growth and neural circuits arrangement and stabilization, thus playing a crucial role in synaptic plasticity. It has been suggested that vitamin D deficiency in the developmental brain could be part of the pathophysiology of schizophrenia through the deregulation of drebrin and GAP-43 [42].

Vitamin D has been shown to upregulate neurotrophic factors, including nerve growth factor (NGF), glial-derived nerve growth factor (GDNF), and neurotrophin 3 (NTF3) [37]. NGF is a pivotal molecule driving neuronal survival of hippocampal and cortical neurons. Gezen-Ak et al. documented that vitamin D regulates NGF release and prevents cytotoxicity in primary hippocampal neuron cultures [43]. GDNF and its receptor proto-oncogene tyrosine-protein kinase receptor Ret (C-Ret) have been recently shown to be directly regulated by vitamin D in SH-SY5Y cells [44]. This result confirms previous findings on the role that vitamin D plays in the differentiation of dopaminergic neurons by influencing critical enzymes involved in dopamine production pathways, such as tyrosine hydroxylase and catechol-O-methyltransferase [45].

Finally, vitamin D helps neuroprotection through several mechanisms. It prevents excitotoxicity injury caused by a sudden increase in cytoplasmic Ca^2+^, up-regulates the synthesis of parvalbumin and calbindin, and down-regulates L-type voltage-gated calcium channels (L-VGCCs) [46]. The hormone exerts anti-inflammatory activities, inhibits the inducible synthesis of nitric oxide (iNO), and increases γ-glutamyl-transpeptidase in glutathione pathways, reducing oxidative burden within neurons and microglia [47,48].

Due to the evidence mentioned above, vitamin D is deemed to contribute to the connectivity of the ventral tegmental area-accumbens nucleus-prefrontal cortex circuit and the nigro-striatal circuit, which are dopaminergic neural circuits involved, respectively, in reward-dependent and motor behavior [49,50]. Further, the influence of vitamin D status in neurocognition has been suggested, also due to the wide presence of VDR and 1α-hydroxylase within the brain areas involved in cognitive processes like complex planning and formation of new memories [51,52].

## 6. Lacking 25(OH)D_3_ Standardized Data: The Case of Alzheimer’s Disease and Parkinson’s Disease

The best biomarker for vitamin D status is 25-hydroxyvitamin D (25(OH)D_3_). Low 25(OH)D_3_ serum levels in neurological, autoimmune and infectious diseases are a common finding [53,54,55,56]. Together with cardiovascular and inflammatory markers, 25(OH)D_3_ has been proposed as a serum biomarker in neurodegenerative disorders [57,58,59,60,61,62,63,64,65]. It has also been proposed as a serum biomarker of disease severity during infections, along with well-established biomarkers [66,67,68,69]. However, there was a lack of standardization in 25(OH)D_3_ measurement procedures and materials in the past, leading to an elusive definition of optimal vitamin D status, as shown for other analytes [4,5,6]. Standardization process aligns laboratories and assays with the “true” 25(OH)D_3_ concentration, based on internationally recognized reference procedures and materials, regardless of the location, time, and system. The Vitamin D Standardization Program (VDSP) was recently funded to reduce total 25(OH)D_3_ measurement analytical variability, encouraging manufacturers and research and routine clinical care laboratories to use methods and materials traceable to NIST RMPs and standard reference materials (SRMs) [6]. However, the lack of standardization of 25(OH)D_3_ measurement has hampered the development of consensus guidelines defining vitamin D deficiency, insufficiency and sufficiency. This yields to the difficulty in interpreting a large amount of literature data available, since the majority of the studies in the field of vitamin D mainly report unstandardized results [6]. This is especially apparent in relation to the studies evaluating 25(OH)D_3_ serum levels in AD. Substantial evidence shows that vitamin D deficiency is associated with cognitive impairment [70,71,72,73], and an association between low 25(OH)D_3_ serum levels and the risk of developing AD has been reported by several authors [54,74,75,76,77]. However, many studies, including those with a long-term follow-up longitudinal design, reported conflicting results [78,79,80]. Discrepancies among the findings can also be explained by variation across the cut-off used to define vitamin D deficiency [43]. A large meta-analysis also reported substantial heterogeneity among the studies reviewed due to differing vitamin D assay methods used [81]. The main concern about most of the studies is that they report unstandardized data, as only a few authors certified the use of internationally recognized procedures and materials. Thus, the role of 25(OH)D_3_ as a serum biomarker in AD remains uncertain, although broad literature data are available.

The same scenario can be observed when evaluating the studies on the role of vitamin D in Parkinson’s disease (PD). Although low 25(OH)D_3_ serum levels have been largely reported among PD patients [82,83,84], it should be noted that the studies reporting the use of certified materials were few and with small sample size [85]. An association between 25(OH)D_3_ serum levels and PD severity have been also reported, but here again the sample size was too small to give strength to study results [86]. Further, the meta-analysis performed in this field should be interpreted with caution due to the high heterogeneity among the assay methods used in the studies reviewed [51]. As in the case of AD, only a few studies evaluating relatively small samples reported standardized data, and meta-analysis are of little use; therefore, available data do not support a role for serum 25(OH)D_3_ in PD.

Liquid chromatography-mass spectrometry (LC-MS) based assays have been developed to measure other metabolites and molecules, including vitamin D2 and vitamin D3 forms of 1,25(OH)2D_3_, 3-epi-25(OH)D_3_, 24,25 (OH)2D3, vitamin D-binding protein (DBP) and free/bioavailable 25(OH)D_3_, to be used as potential biomarkers for vitamin D status [87]. NIST has also developed RMPs for 25(OH)D2, and 24R,25(OH)2D_3_, and disseminated serum-based SRMs with values assigned for 25(OH)D2, 25(OH)D_3_, 3-epi-25(OH)D_3_, and 24R,25(OH)2D_3_ [88]. However, their usefulness in AD and PD has not been thoroughly evaluated yet.

## 7. Summary and Conclusions

Vitamin D plays a key role in various physiological processes, ranging from the modulation of the immune response to the regulation of brain development and activities in adulthood. Hence, vitamin D has been long studied in many pathological conditions, either as a risk factor or a serum biomarker for disease severity. Unfortunately, the lack of 25(OH)D_3_ measurement standardization in the past hampered the development of consensus guidelines defining vitamin D deficiency, insufficiency, and sufficiency, thus leading to the difficulty in interpreting a considerable volume of literature data available. This is the case for AD and PD, for which vitamin D could represent a good candidate as a serum biomarker, but, despite a growing body of literature data, the usefulness of the studies is weakened by the discrepancies in the assay methods and cut-offs used. Standardized data are required to perform meaningful meta-analysis, supporting reliable conclusions on the potential role of vitamin D in AD and PD.
